# Thermal stability of Si/SiC/ta-C composite coatings and improvement of tribological properties through high-temperature annealing

**DOI:** 10.1038/s41598-022-07514-8

**Published:** 2022-03-03

**Authors:** Young-Jun Jang, Jae-Il Kim, Won-seok Kim, Do Hyun Kim, Jongkuk Kim

**Affiliations:** 1 Department of Extreme Environmental Coatings, Extreme Materials Institute, Korea Institute of Materials Science, 797, Changwon-daero, Seongsan-gu, Changwon-si, Gyeongsangnam-do 51508 Republic of Korea; 2 Department of Micro-Nano Mechanical Science and Engineering, Graduate School of Engineering, Nagoya University, Furo-cho, Chikusa-ku, Nagoya, Aichi 464-8603 Japan

**Keywords:** Materials science, Engineering, Mechanical engineering

## Abstract

We report the structure, mechanical properties, thermal stability, and durability of Si/SiC/ta-C composite (Si–ta-C) coatings fabricated using simultaneous filtered cathodic vacuum arc deposition and direct current unbalanced magnetron sputtering. Si concentration of 1.25–6.04 at.% was achieved by increasing the unbalanced magnetron sputtering power from 25 to 175 W. Si addition provided functionality to the coating, such as heat resistance, while retaining the high hardness of ta-C coatings. The Si–ta-C coatings were stable up to 600 °C regardless of the Si content, while the coating containing 3.85 at.% Si was stable up to 700 °C. The friction behavior and mechanical properties were dependent on the coating film before and after annealing at 100–200 °C; however, annealing at 300–400 °C decreased disk wear and increased counterpart wear due to an increase in film hardness on account of an endothermic reaction that increased the number of Si–C bonds. This indicates that the basic hardness characteristics of the ta-C coating and the high-temperature structural change of the Si–ta-C coating are important for ensuring high-temperature durability. These characteristics were verified through the low coefficient of friction and wear rate of the 1.25 at.% Si–ta-C coating after annealing at 500 °C.

## Introduction

Diamond-like carbon (DLC) coatings have excellent mechanical and chemical properties and have been applied in many industrial fields. In order to increase the reliability and durability on an optical mold, a DLC coating having low friction properties and high-temperature durability properties at the same time is attracting attention^1^. Conventionally, a glass mold press process at ~ 100 to 300 °C has been performed, however, a heat resistance under temperature of 300 °C or more is recently required with a development of glass materials. When operated above 300 °C in air, hydrogenated DLC coatings applied to moving parts experience rapid graphitization—whereby the sp^3^ phase is converted to an sp^2^ phase—owing to their low thermal stability characteristics^2–6^. In addition, the hydrogen in hydrogenated DLC films causes the hardness to degrade after exposure to elevated temperatures. On the other hand, non-hydrogenated DLC coatings such as tetrahedral amorphous carbon (ta-C) are stable up to 500 °C owing to their high sp^3^ phase fraction^5,7,8^. However, the heat resistance of non-hydrogenated DLC coatings is still too low for moving parts operated in high-temperature environments under external forces. According to Deng et al.^9^, ta-C coatings can easily peel off a substrate in high-temperature (500 °C) ambient air, exposing the substrate.

Thus, the graphitization of both non-hydrogenated and hydrogenated DLC coatings starts within the range of 300–400 °C^10^. The graphitization behavior was described by Wang et al.^11^, and can be explained based on the oxidation behavior of DLC coatings^11,12^. Amorphous DLC coatings undergo crystallization, graphitization, carbonization, and structural changes when the temperature approaches 350 °C. In addition, the weight loss of the DLC coating is reduced between 350 and 450 °C owing to oxidation via an endothermic reaction.

To suppress the endothermic reaction, transition metals such as Si can be introduced into the DLC coating. This improves the thermal stability and high-temperature tribological behavior. However, increasing the Si content can reduce the durability owing to a decrease in the mechanical properties. Si co-deposition is typically undertaken during the DLC coating procedure. Representative DLC coating methods include sputtering and C_2_H_2_ and CF_4_ gas-based chemical vapor deposition (CVD)^13–18^. The mechanical properties of CVD-based Si-doped DLC coatings are reported to be between 12 and 15 GPa, while those of Si-doped DLC coatings prepared by hallow-cathode plasma immersion ion implantation reach up to 22 GPa^13^. In addition, the mechanical and tribological properties of CVD-based Si-doped DLC coatings deposited using tetramethylsilane (TMS, Si(CH_3_)_4_) have been found to decrease at temperatures above ~ 400 °C^2^. These issues must be addressed; however, despite interest in high-temperature tribological behavior, few studies have considered methods of preventing the thermal degradation of Si-doped DLC coatings.

Herein, we adopt ta-C as a non-hydrogenated DLC coating and attempt to reduce the thermal degradation while maintaining high mechanical properties—one of the main advantages of ta-C coatings—by Si incorporation (1.25–6.04 at.% Si). The composite coating was prepared by a filtered cathode vacuum arc (FCVA) deposition method with unbalanced magnetron (UBM) sputtering. This study provides new insights into improving the thermal stability and tribological properties of Si/SiC/ta-C composite (Si–ta-C) coatings by elucidating the effect of Si co-deposition and annealing on the structure, mechanical properties, heat resistance, and tribological characteristics of ta-C coatings at elevated temperatures.

## Materials and methods

### Deposition of ta-C and Si–ta-C coatings

**Figure 1:**
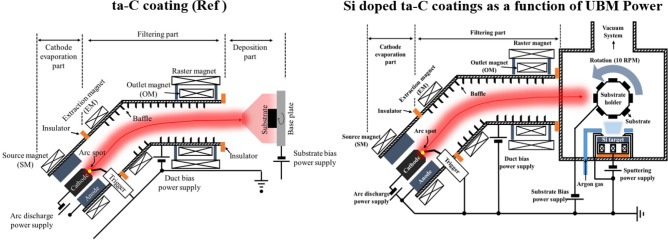
Schematic illustration of tetrahedral amorphous carbon (ta-C) and Si/SiC/ta-C composite (Si–ta-C) coating procedures using a filtered cathode vacuum arc (FCVA) and unbalanced magnetron (UBM) sputtering coating system.

A standard ta-C coating was deposited on a tungsten-carbide cobalt substrate (WC–Co, 10 × 10 × 5 mm), which is mainly utilized as a mold material in GMP process, using a 45°-type filter in an FCVA coating system. The total thickness of the coating film was 750 nm, comprising a 250-nm-thick Ti interlayer and a 500-nm-thick ta-C coating layer. A Ti layer was inserted as an interlayer to improve the adhesion between the substrate, and ta-C and to prevent any possible graphitization induced thermal decomposition of carbide bond with Co. Weak carbide-forming metals such as Co lead to result in Csp^2^ bonds due to thermal decomposition at high temperatures^19–21^. A description of the equipment is provided in Ref.^22^. To increase the thermal stability and durability of the coating film, UBM sputtering (Ar gas flow: 15 sccm) was simultaneously discharged during ta-C coating to form a Si–ta-C coating. Figure 1 shows a schematic illustration of the ta-C and Si–ta-C coating systems. The deposition conditions of the ta-C coating film were as follows: duct bias, 10 V; arc current, 60 A; substrate bias, − 30 V; and working pressure, 3 × 10^−2^ Pa.

**Figure 2:**
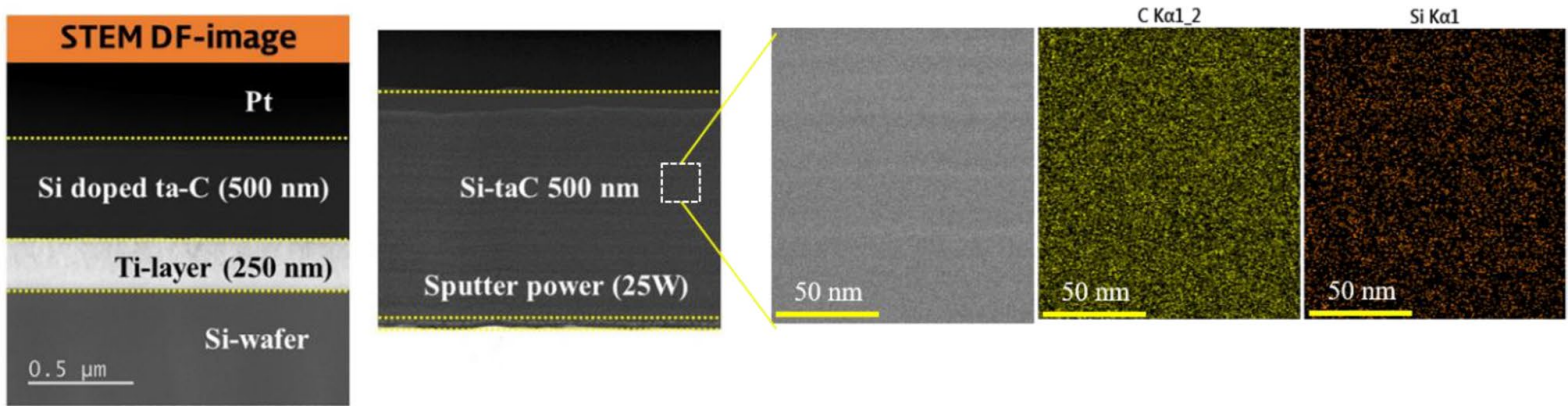
Scanning transmission electron microscopy (STEM) Dark Field (DF) images and energy dispersive X-ray spectroscopy (EDS) data of Si/SiC/ta-C composite (Si–ta-C) coating with a representative sputtering power of 25 W (indicated by white-dashed square). 1.25 at.% Si/SiC/ta-C composite deposited on Si-wafer and was prepared by cross-sectional cutting through focused ion beam.

The Si co-deposition ratio was controlled by changing the direct current (DC) sputtering power (25, 75, 125, and 175 W). To exclude variables that change with the coating thickness, the thickness of the Si–ta-C coating films was maintained at 500 nm by monitoring the change in the Si deposition rate at each sputtering power. The coating structures was observed using field-emission transmission electron microscopy (FE-TEM; JEOL JEM-ARM200F, USA) with an acceleration voltage of 200 kV. Cross-sectional scanning transmission electron microscopy (STEM) and energy dispersive X-ray spectroscopy (EDS) images of the Si–ta-C coating produced at a sputtering power of 25 W are shown in Fig. 2 (Section 1 in supplementary materials).

The 250-nm-thick Ti interlayer and 500-nm-thick Si-ta-C coating were successfully deposited by the simultaneous FCVA/UBM sputtering system. In addition, the C component was successfully co-deposited with Si.

### Surface characterizations

The prepared samples were annealed in an electric furnace (World system-WS-HF-130406, South Korea) in air at target temperatures of 100, 200, 300, 400, 500, 600, and 700 °C. The furnace temperature was increased to the target temperature, held for 1 h, and then slowly cooled to room temperature. The coatings were then observed using laser confocal microscopy (OLS5000; Olympus, Japan) to analyze the thermal stability characteristics and extent of oxidation after annealing.

X-ray photoelectron spectroscopy (XPS; Thermo Fisher Scientific NEXSA, USA) with Al Kα radiation aided the chemical bonding state analysis and sp^2^/sp^3^ characterization of the ta-C and Si-ta-C coating films. XPS analysis of the carbon bonding states was conducted by performing a “Shirley” background subtraction procedure^23^. The binding energies of C(sp^2^), C(sp^3^), C–O, C=O, and Si–C were 284.5, 285.3, 286.6, 288.5, and 283.6 eV, respectively, and fitting was performed using four Voigt function Gaussian (80%) and Lorentzian (20%) models^24^. Fitting for the XPS analysis was conducted using Origin 2017 with a tolerance of 1 × 10^−9^ and 400 iterations^25,26^.

Fourier transform infrared (FT-IR) spectroscopy (Spectrum two; PerkinElmer, USA) and Raman spectroscopy (Horiba Co. Ltd., Japan; *λ* = 532 nm) aided the structural analysis of the ta-C coating film. The laser power, spot size, and integration time were 100 mW, 2 µm, and 1–10 s, respectively, and five measurements were conducted on each sample. A nano-indenter (NHT3; Anton Paar, Austria) was used to measure the hardness and elastic modulus. The average indentation load was 10 mN, and the average value was calculated by measuring each sample ten times. The Poisson’s ratio was taken as 0.180. The change in structural analysis was determined by X-ray diffraction (XRD; PANalytical Xpert Pro MRD, Netherland) with Cu Kα radiation at 40 kV and 30 mA in the 2θ range of 20°–80°.

### Ball-on-disk type tribo-test in air

The tribological characteristics of the annealed ta-C coatings were examined using a ball-on-disk tribometer with a Si_3_N_4_ ball (diameter: 8 mm, Vickers hardness: 15 GPa^27^) as the counterpart. The normal load was 2 N, and the rotation speed was 200 rpm with a 4.5 mm wear track radius. A total of 10,000 sliding cycles were performed. All experiments were conducted at a temperature of 26 °C and 60% relative humidity in ambient air. After performing the tests, the wear rate of the film was calculated based on the profile of the wear track, which was measured using laser confocal microscopy. The wear volume of each wear track was obtained as the average of five measurements in randomly selected areas.

## Results and discussion

### Structural and mechanical characterizations as a function of Si concentration

**Figure 3:**
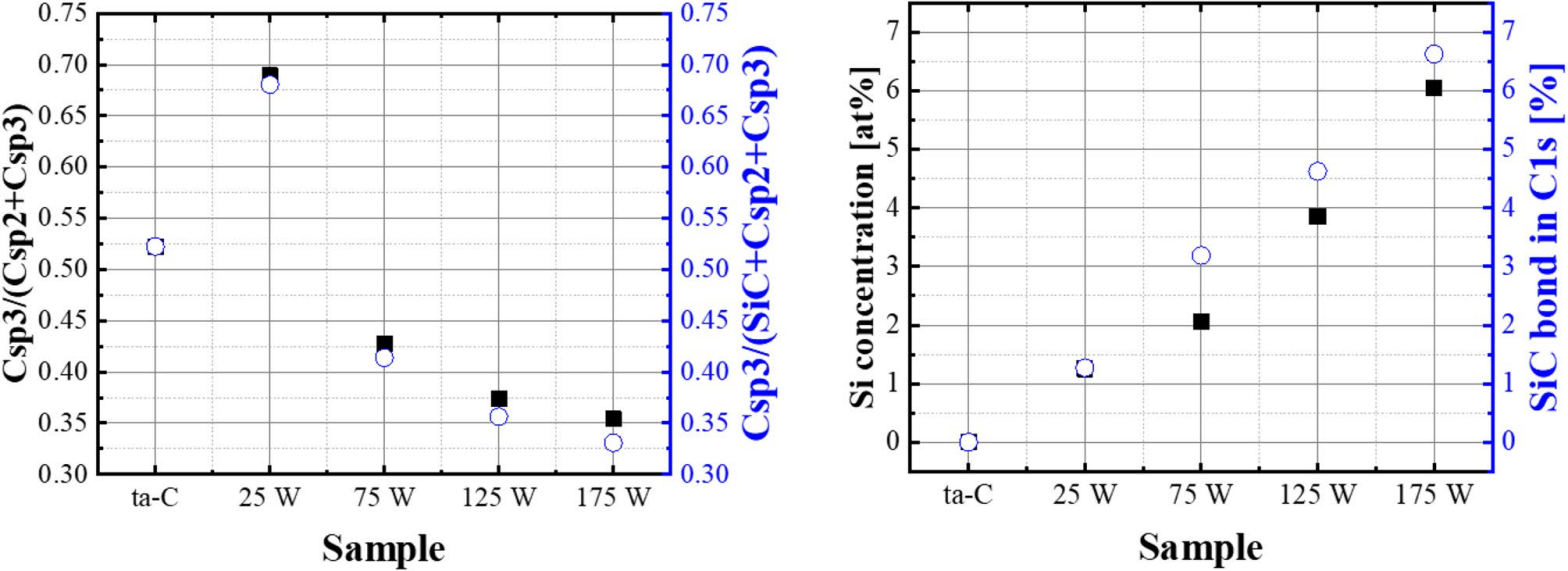
Behavior of C(sp^3^)/C(sp^2^) ratio and Si atomic percent and SiC bond percent of tetrahedral amorphous carbon (ta-C) and Si/SiC/ta-C composite (Si–ta-C) coatings as a function of unbalanced magnetron (UBM) sputtering power from the X-ray photoelectron spectroscopy (XPS) C 1 s spectra.

The XPS spectra of the ta-C and Si–ta-C coatings were analyzed for structural analysis and sp^2^/sp^3^ characterization. Fitting of the C 1 s spectra revealed the presence of C(sp^3^) (285.3 eV), C(sp^2^) (284.5 eV), CO (286.6 eV), a contaminant phase, and SiC (283.6 eV). The sp^3^ content was calculated as the ratio of the total area of the sp^2^, sp^3^, C–O, C=O, and Si–C peaks to the area of the sp^3^ peak^28^. Figure 3 shows the results of sp^3^ bonding, Si concentration, and SiC bonding in the C 1 s peak as a function of sputtering power (Section 2 in supplementary materials), and Table 1 shows the sp^3^ fraction ratio, Si, and Si–C bond ratio as a function of sputtering power. The sp^3^ fraction of the ta-C coating film was 52%. For the Si–ta-C coatings (sputtering power of 25–175 W), the sp^3^ fraction was highest with a sputtering power of 25 W (sp^3^ fraction = 69%), and then decreased as the sputtering power was increased further (sp^3^ fraction at a sputtering power of 175 W = 36%).

**Table 1:** sp^3^ content, Si atomic ratio in survey spectra, and Si–C fraction in C1s spectra of the tetrahedral amorphous carbon (ta-C) and Si/SiC/ta-C composite (Si–ta-C) coatings as a function of sputtering power during ta-C deposition using XPS.

Samples	Sputtering power (W)	sp^3^ content (%)	Si (at.%)	Si–C fraction (%)
ta-C	0	52	–	–
Si–ta-C	25	68	1.25	1.27
Si–ta-C	75	41	2.06	3.19
Si–ta-C	125	37	3.86	4.63
Si–ta-C	175	33	6.04	6.63

Through XPS analysis, it was observed that both the Si atomic percent and Si–C bond percent increased with sputtering power. This correlates with the reduction in sp^3^ fraction with increasing sputtering power. Wan et al. reported that at low silicon concentration, the sp^3^ fraction for interlinks of C atoms increases^29^. On the other hand, at a high silicon contents level, Si incorporation reduce the sp^3^ fraction of interlinks of DLC by substituting C atoms in the sp^3^ matrix. Lee et al.^30^ reported, that for a sputtered Si–ta-C coating, when the Si concentration is less than 2.5 at.%, most of the Si atoms that substitute the C atoms of the sp^3^ bonds are likely to be isolated from each other. Because Si–C bonds are weaker than sp^3^ C–C bonds, the Si-incorporated sites can assist with compensating the distortion of the nearby sp^3^ C–C bonds. These reports regarding effects on Si concentration provide support to our finding, and the results are in agreement with previous arguments. Herein, when the sputtering power was 25 W, the ratio of Si atomic percent to Si–C bond percent in the ta-C coating film was 1:1, as shown in Fig. 3b. Thereafter, this ratio increased as the sputtering power increased. In particular, the Si–C ratio was higher from a sputtering power of 75 W onward.

**Figure 4:**
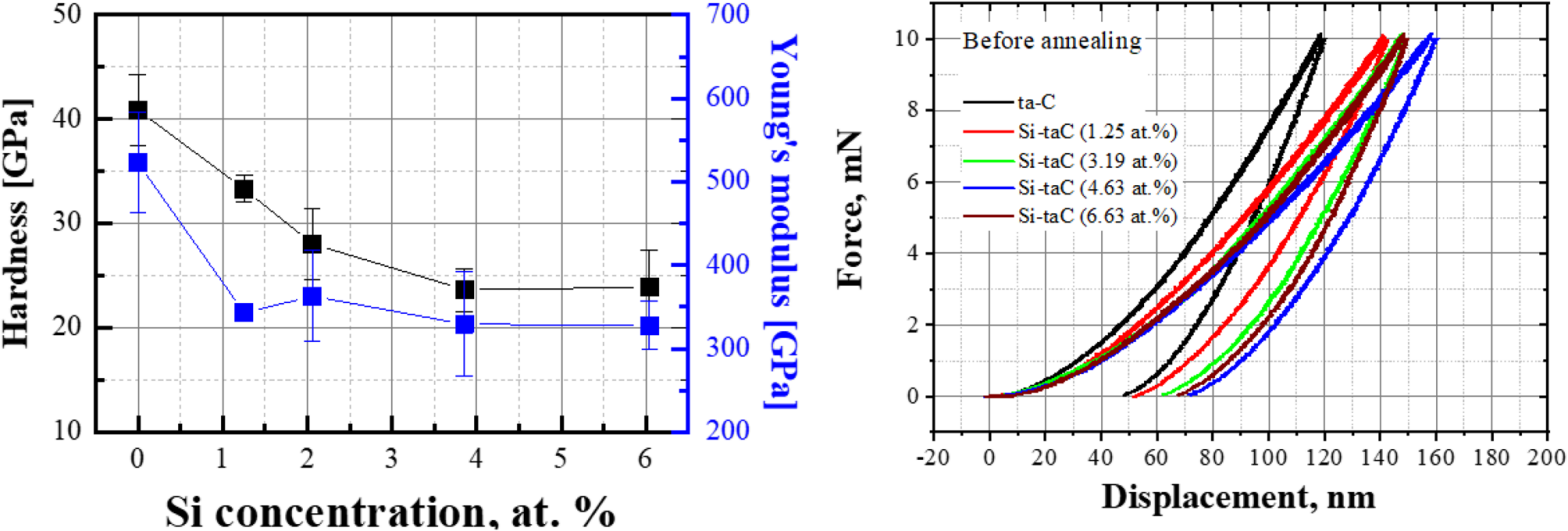
Hardness and elastic modulus at 10 mN of the normal force for tetrahedral amorphous carbon (ta-C) and Si/SiC/ta-C composite (Si–ta-C) coatings as a function of Si concentration (measured using nano-indenter).

As the Si concentration increased, the mechanical properties of the coatings decreased. Figure 4 shows the hardness (*H*) and elastic modulus (*E*) as functions of Si concentration. The hardness and elastic modulus of the ta-C coating were measured to be 41 and 522 GPa, respectively. However, as the Si concentration increased from 1.25 to 3.85 at.%, the hardness decreased from 33 to 24 GPa. No further decrease in either the hardness (~ 23 GPa) or elastic modulus (~ 328 GPa) was observed when the Si content increased from 3.85 and 6.04 at.%. The hardness and elastic modulus of silicon carbide are approximately 27 and 315 GPa^31^; therefore, the maintenance of mechanical properties at higher Si concentrations is likely due to an increase in Si–C bonding. Based on these results, the mechanical properties can be divided into a low Si concentration region (region 1; 1.25–3.85 at.% Si) and a high Si concentration region (region 2; 3.85–6.04 at.% Si).

### Thermal stability of the ta-C and Si–ta-C coatings as a function of Si concentration

**Figure 5:**
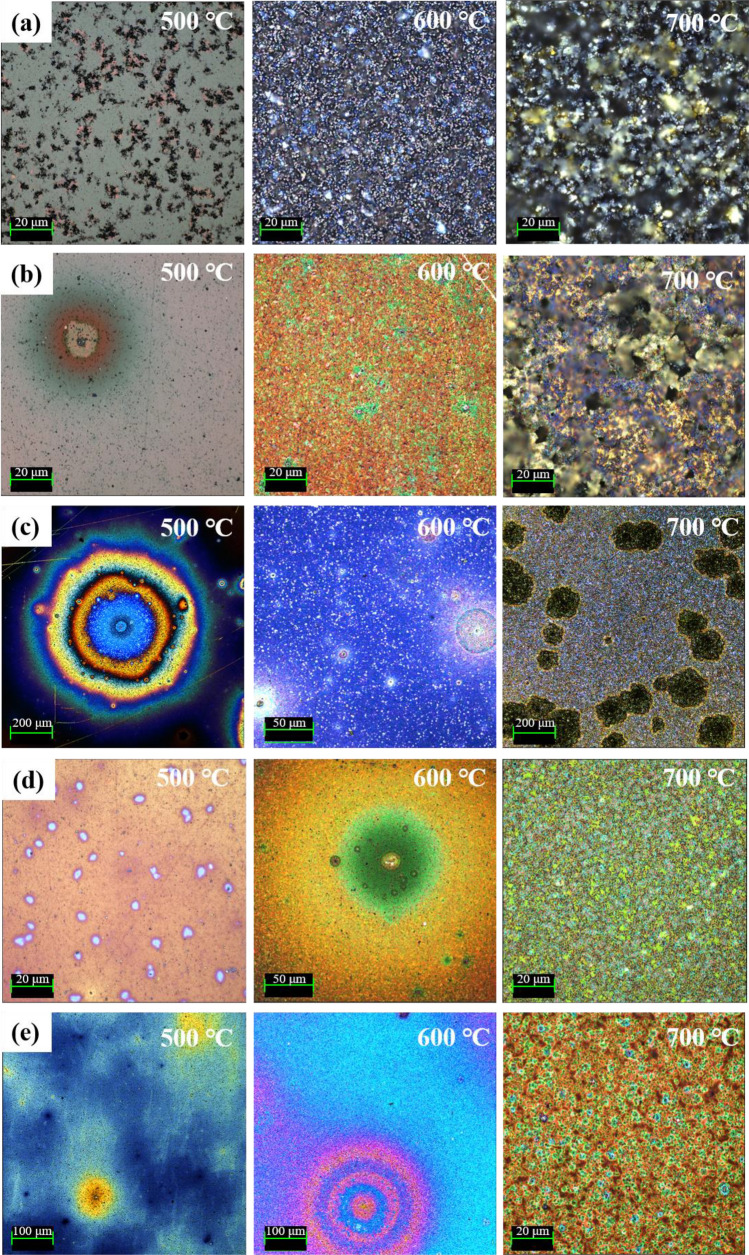
Surface images of tetrahedral amorphous carbon (ta-C) and Si/SiC/ta-C composite (Si–ta-C) coatings annealed at 500, 600**,** and 700 °C: (**a**) ta-C coating, (**b**) 1.25 at.%, (**c**) 2.06 at.%, (**d**) 3.86 at.%, and (**e**) 6.04 at.% Si–ta-C coatings.

The thermal degradation behavior of the ta-C and Si–ta-C coatings was analyzed by surface observations and Raman analysis after annealing for 1 h in air. When a coating film undergoes high-temperature oxidation, it is common for the weight of the coating film to be reduced. One unique phenomenon of ta-C coatings is that the surface color changes in the visible region as the coating thickness is changed, because of defects with a graphitic structure on the ta-C coating surface^32^. Figure 5 shows surface micrographs of the coating films after annealing at different temperatures. In the case of the ta-C coating (Fig. 5a), partial exposure of the substrate (WC–Co) was observed after annealing at 500 °C, indicating that the coating film underwent carbonization. After annealing at higher temperatures, irregular agglomeration of the WC–Co substrate and complete removal of the coating film were observed.

**Figure 6:**
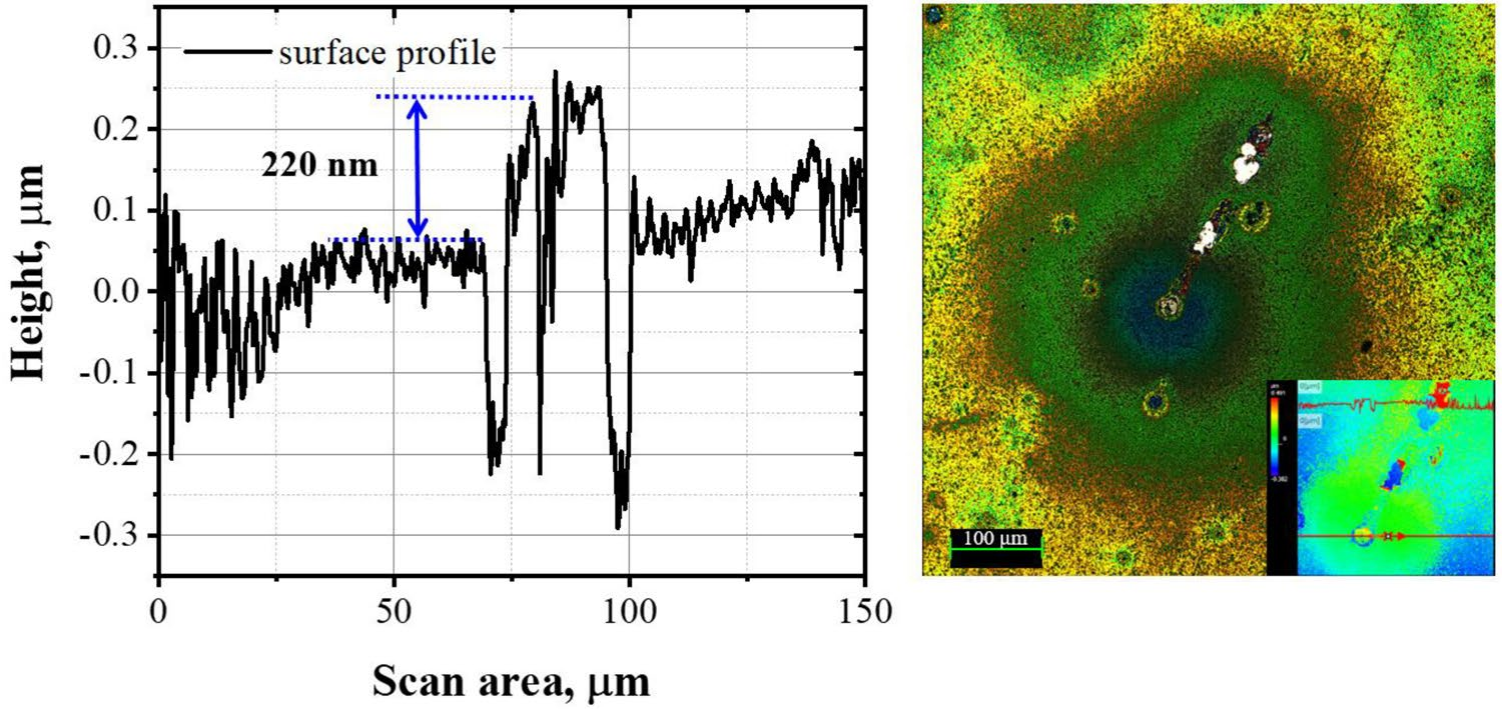
Residual thickness of Si/SiC/ta-C composite (Si–ta-C) coating (6.04 at.% Si) after annealing at 700 °C.

In contrast, the Si–ta-C coatings (Figs. 5b–e) were stable up to 600 °C, regardless of the Si concentration. Moreover, the thermal stability at 700 °C increased as the Si concentration increased. At a low Si concentration of < 2.00 at.%, annealing at 700 °C resulted in carbonization around the defects on the surface, and ta-C was lost. In contrast, at a high Si concentration of > 3.86 at.%, the Si–ta-C coating surface was maintained, despite some weight loss due to high-temperature oxidation. In particular, the coating with 6.04 at.% Si had a residual thickness of 220 nm after annealing for 1 h at 700 °C (original Si–ta-C thickness: 500 nm), which indicates excellent relative thermal stability of the coating film. The residual thickness of the coating was measured around the surface defects after high-temperature oxidation. Figure 6 shows the residual thickness of the Si–ta-C (3.86 at.% Si) coating after annealing at 700 °C.

**Figure 7:**
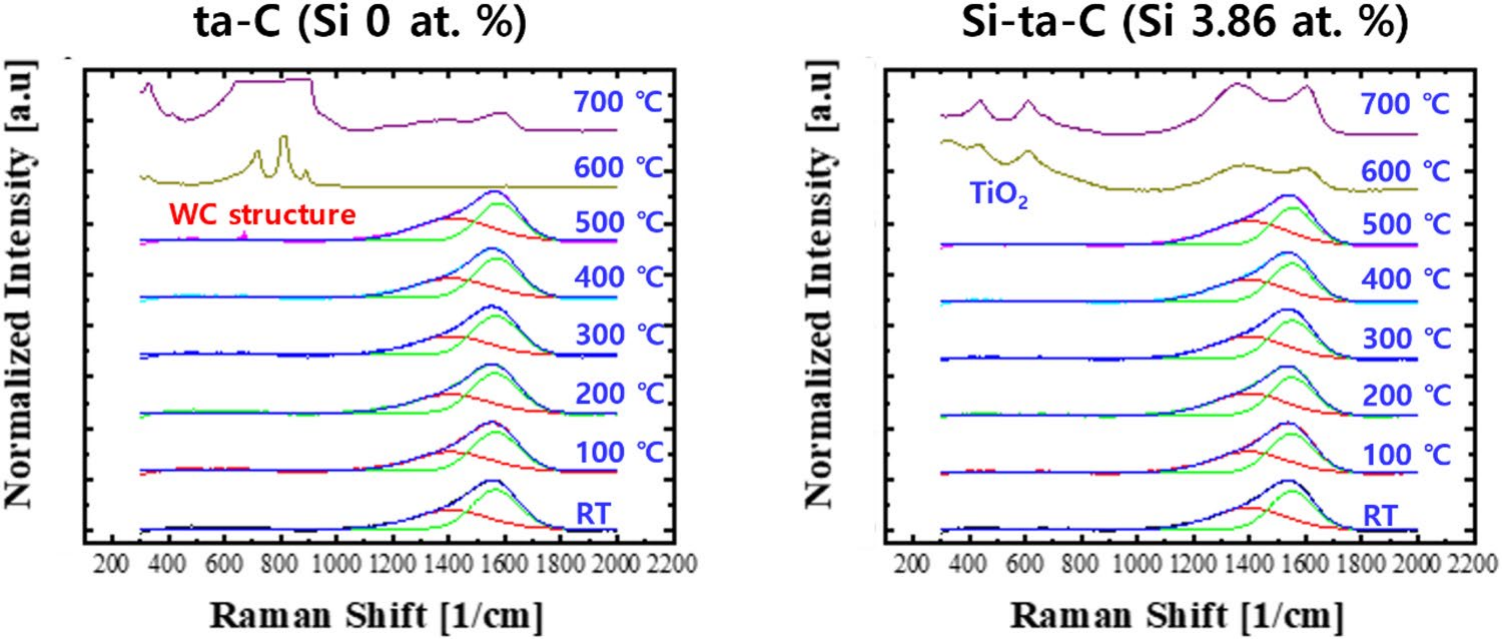
Raman spectra of tetrahedral amorphous carbon (ta-C) and 3.86 at.% Si-incorporated ta-C (Si–ta-C) coatings before and after annealing at elevated temperature.

The thermal degradation of the ta-C and Si–ta-C (3.86 at.% Si) coatings after annealing was examined using Raman spectroscopy, as shown in Fig. 7. Peak analysis revealed no changes in the shapes of the peaks after annealing at temperatures of up to 400 °C. The partial weight loss of the ta-C coating layer and Ti interlayer caused by high-temperature oxidation and the exposure of the WC–Co substrate (709, 806 cm^−1^)^33^ were first observed after annealing at 500 °C. In addition, at 600 and 700 °C, complete removal of the ta-C coating layer was confirmed, and peaks were observed for the WC substrate. Moreover, a large number of uninterpretable impurities appeared. However, in the case of the Si-ta-C coating, only a minimal change was observed in the shape of the Raman spectra up to 500 °C. At 600 °C, TiO_2_ (440, 610 cm^−1^)^34^ peaks were generated simultaneously with the weight loss of Si-ta-C coating. Even at 700 °C, a structural change occurred owing to accelerated graphitization, and the TiO_2_ peak was observed in a mixed state. Therefore, the thermal stability of the ta-C and Si–ta-C (3.86 at.% Si) coating films was confirmed through surface and Raman analysis. In particular, heat resistance characteristics were well established on account of the residual thickness of the Si–ta-C (3.86 at.% Si) coating after annealing at 700 °C.

**Figure 8:**
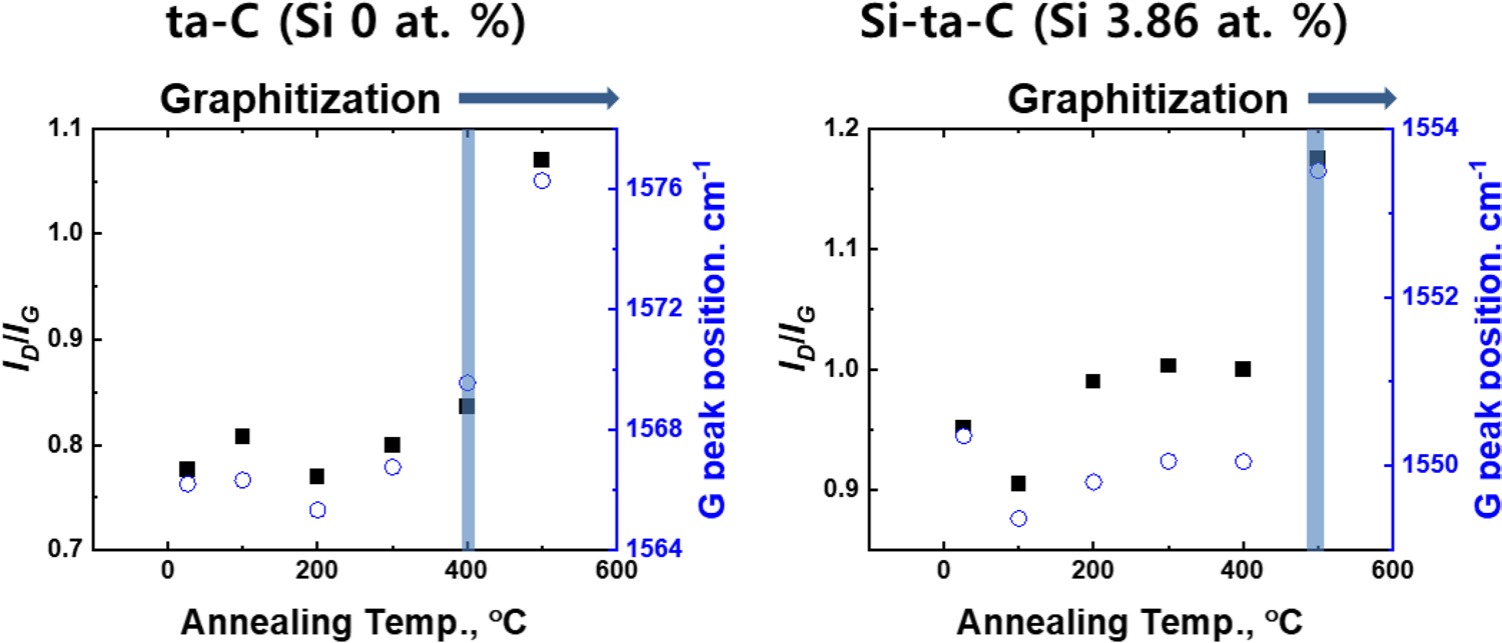
*I*_D_/*I*_G_ ratio and G peak position of tetrahedral amorphous carbon (ta-C) and 3.86 at.% Si-incorporated ta-C (Si–ta-C) coatings before and after annealing at different temperatures (analyzed using Raman spectroscopy).

Thermal degradation of the carbon network was confirmed by analyzing the *I*_*D*_/*I*_*G*_ ratio and G peak position (Fig. 8). For ta-C, a change in the G peak position was detected at 400 °C, whereas for Si–ta-C (3.86 at.% Si), it was detected from 500 °C. Thus, Si co-deposition improves the thermal degradation resistance. The change in *I*_*D*_/*I*_*G*_ ratio further supported this result.

This proves that an appropriate Si concentration should be included to reinforce the high-temperature stability and durability.

The thermal stability characteristics were improved by Si co-deposition. In addition, the inclusion of a small amount of Si resulted in a relatively small decrease in hardness compared to that of high amount of Si; this helps to verify the high-temperature durability.

**Figure 9:**
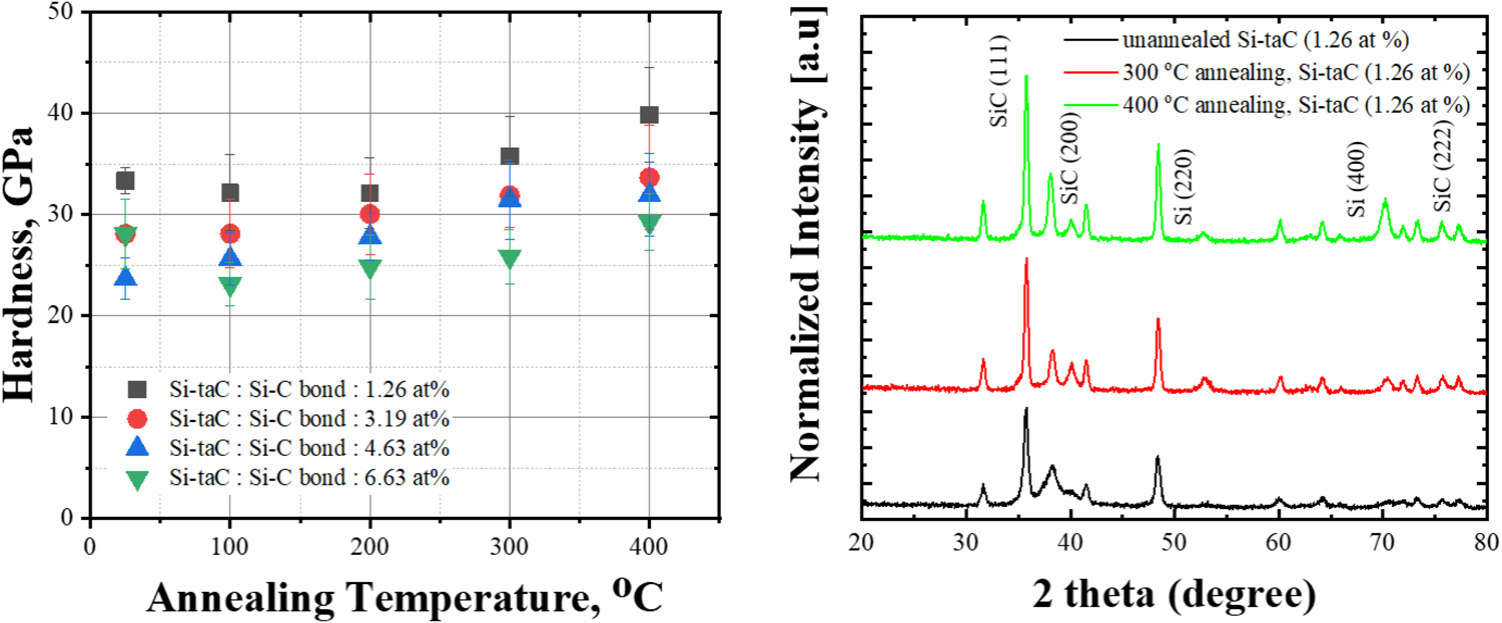
Hardness of Si/SiC/ta-C composite (Si–ta-C) coatings after annealing at different temperatures and X-ray diffraction (XRD) patterns of Si-ta-C (1.26 at.% Si) before and after annealing at 300 and 400 °C.

Nanoindentation was used to investigate how the mechanical properties changed after exposure to elevated temperatures. Figure 9 shows the hardness of the Si-ta-C coatings before and after annealing at 100–400 °C (Section 3 in supplementary materials). The hardness of the Si–ta-C coating films increased with annealing temperature. In particular, the hardness of the low Si film (1.25 at.% Si) increased up to 40 GPa after annealing at 400 °C. In the tribological tests, this led to a decrease in disk wear and increase in counterpart wear, as discussed in Sect. 3.3. It is likely that this increase in hardness occurred owing to an increase in the crystallization of SiC phase in the coating film on account of the endothermic reaction of C as the annealing temperature increased to 300 °C.

Figure 9 shows XRD patterns of the Si–ta-C (1.25 at.% Si) coating before and after annealing at 300 and 400 °C. The XRD pattern of the as-deposited film contained SiC(111), SiC(220), and Si(220) peaks. After annealing at 300 and 400 °C, the intensities of the SiC(111) and Si(220) peaks increased, and SiC(200), SiC(222), and Si(400) peaks appeared. These results confirm the hypothesis that the increase in hardness after annealing is caused by an increase in Si–C bonding. This increase in hardness after annealing is unexpected compared with the thermal degradation of conventional Si-doped hydrogenated DLC coatings; that is, the hydrogen bonds in conventional Si-doped DLC coatings are degraded at elevated temperatures.

**Figure 10:**
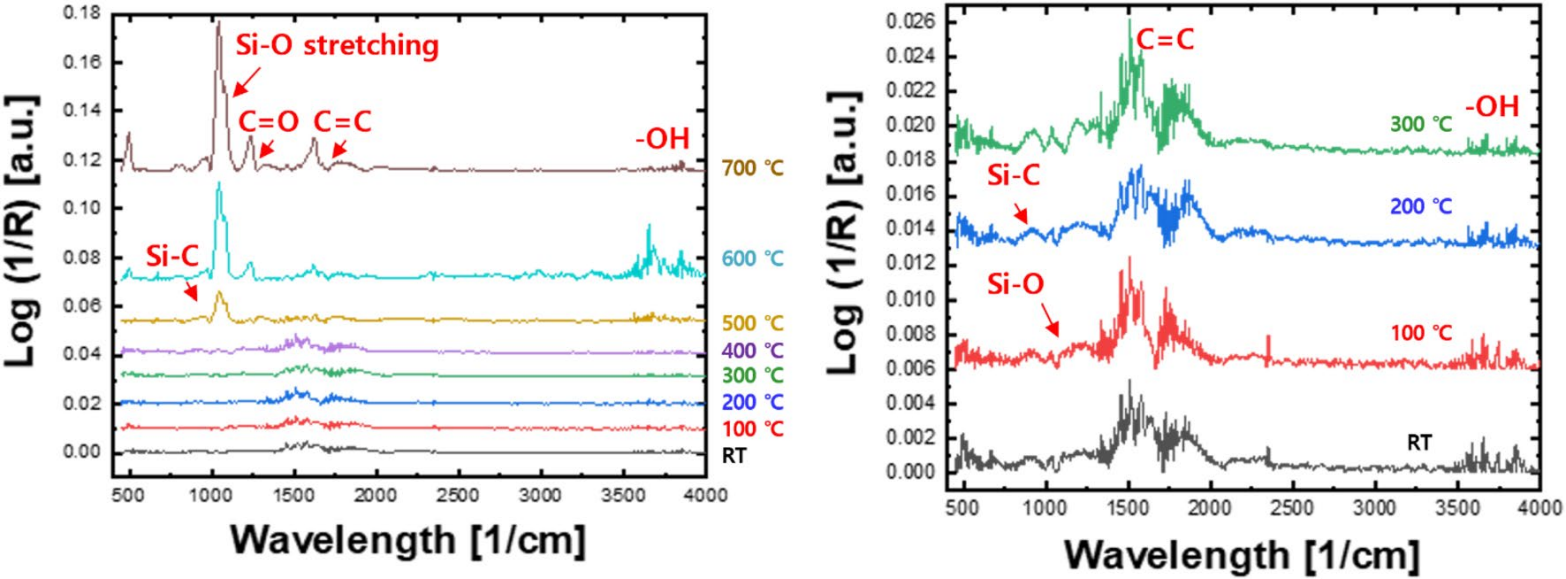
Fourier-transform infrared (FT-IR) spectra of Si/SiC/ta-C composite (Si–ta-C) (3.86 at.% Si) coatings before and after annealing at different temperatures.

Figure 10 shows representative FT-IR spectra of the Si–ta-C coating (3.85 at.% Si). Si–O stretching absorption bands were observed at 1000–1100 cm^−1^^35^. For the Si–ta-C coating film, the intensity of the SiO stretching peak and SiO absorption band increased as the annealing temperature increased. Hence, a SiO network formed during high-temperature annealing; the SiO bonds in the internal structure of the coating film made a substantial contribution to prevent gasification of ta-C.

It is known that high-temperature degradation of ta-C is caused by carbon oxidation and oxygen diffusion, resulting in gasification and graphitization owing to the formation of CO and CO_2_^36–38^. In several reports, it was inferred that the increase in thermal stability in Si-doped DLC was achieved by the oxygen diffusion barrier due to SiO_2_ or Si–O bonding, which gave results similar to our findings^39–41^. Therefore, the oxygen diffusion barrier of the SiO_2_ or Si–O network would have prevented the oxidation-induced gasification of ta-C.

### Friction and wear properties of the ta-C and Si–ta-C coating as a function of Si concentration

Based on the thermal stability results, tribological tests were conducted using as-deposited and annealed samples (annealing temperatures of 100–500 °C). Friction and wear analysis was conducted by splitting the thermal stability results into a range of ~ 400 °C (period 1) and 500 °C (period 2) according to the increase in annealing temperature.

**Figure 11:**
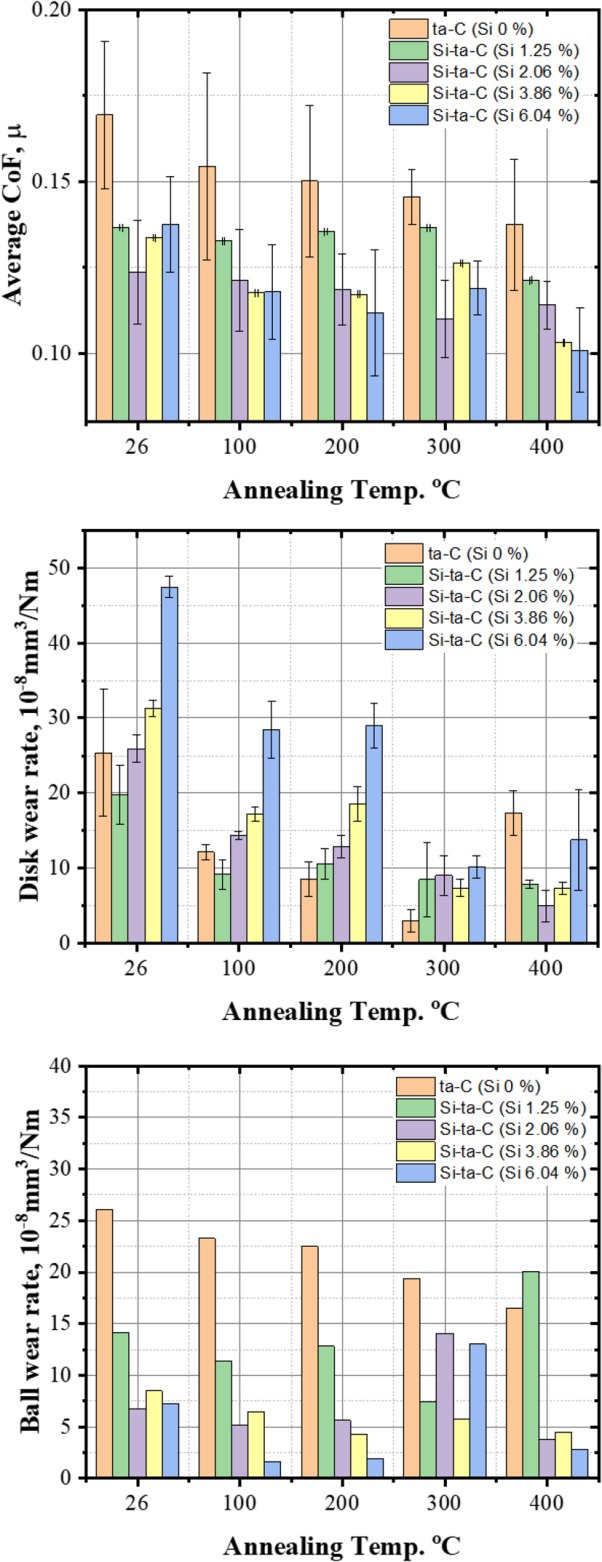
Average coefficient of friction (CoF), wear rate of the disk, and Si_3_N_4_ ball wear rate of tetrahedral amorphous carbon (ta-C) and Si/SiC/ta-C composite (Si–ta-C) coatings before and after annealing at different temperatures (100–400 °C**)** (data derived from analysis of the worn surfaces).

Figure 11 shows the average coefficient of friction (CoF) and disk and Si_3_N_4_ ball wear rates for period 1 (Section 4 in supplementary materials). The average CoF was higher for the ta-C coating than the Si–ta-C coating. As the annealing temperature increased, the CoF decreased for both ta-C and Si–ta-C. This can be attributed to graphitization of the outermost surface of the coating. The endothermic reaction at elevated temperatures caused graphitization and weight loss of the ta-C and Si–ta-C coatings. For the Si–ta-C coatings, low friction was achieved owing to the formation of SiO bonds, which reacted with moisture in the air to form SiO_*x*_(OH)_*y*_ by low energy van der Waals interactions, as expressed in Eq. (1)

^42^.

1 $$ {\text{SiO }} + {\text{ H}}_{{2}} {\text{O }}\left( {{\text{moisture}}} \right) \, \to {\text{ SiO}}x\left( {{\text{OH}}} \right)y $$

The increase in the SiO and SiC network was further activated by high-temperature annealing, as expressed in Eq. (2), resulting in a lower CoF.

2 $$ {\text{Si}} - {\text{ta}} - {\text{C }} + {\text{ O}}_{{2}} \to {\text{ SiO }} + {\text{ SiC}}. $$

This implies that the activation of SiC bonding, owing to the increase in Si content after high-temperature annealing, leads to an increase in the SiO network and therefore in the mechanical properties. This then causes a decrease in the CoF owing to a decrease in the actual contact area, which is a minor parameter that affects the CoF. Thus, as the annealing temperature increases, the increase in mechanical properties owing to activation of the SiC bonds results in a decrease in the actual interfacial contact area.

**Figure 12:**
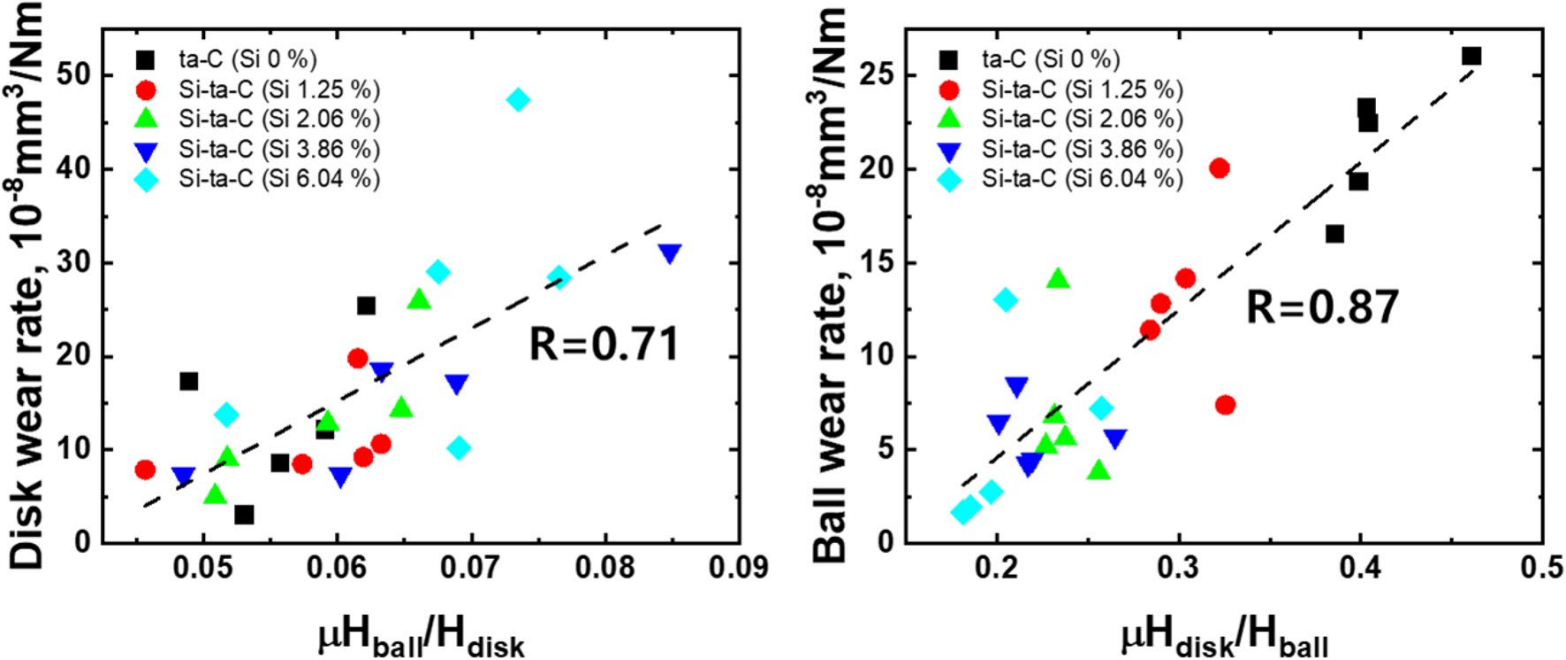
Relationship between abrasive wear index *μH*_disk_/*H*_ball_ (calculated by average CoF and nano-hardness) and disk wear rate and ball wear rate.

A material’s resistance to abrasive wear is related to its hardness and that of its counterpart. The wear rate of the disk and ball also decreased after high-temperature annealing of the ta-C film. This may be due to the response to low shear stress owing to the decrease in the CoF (Fig. 12). Subsequently, the wear rate rapidly increased after annealing at 400 °C, because weight loss of the ta-C coating occurs at elevated temperatures (300–400 °C) owing to the endothermic reaction. Therefore, the low friction factor caused by graphitization is insufficient, and the wear rapidly increases.

However, when the annealing temperature was higher than 300 °C, the wear rate of the Si–ta-C-coated disk considerably decreased, and that of its counterpart increased. This increase in counterpart wear was induced by the increased hardness of the disk. This was clearly observed when the Si content was 1.25 at.%. Therefore, disk wear decreased and counterpart wear increased owing to the strengthening of the mechanical properties of the Si–ta-C coating as a result of thermally induced SiC formation.

**Figure 13:**
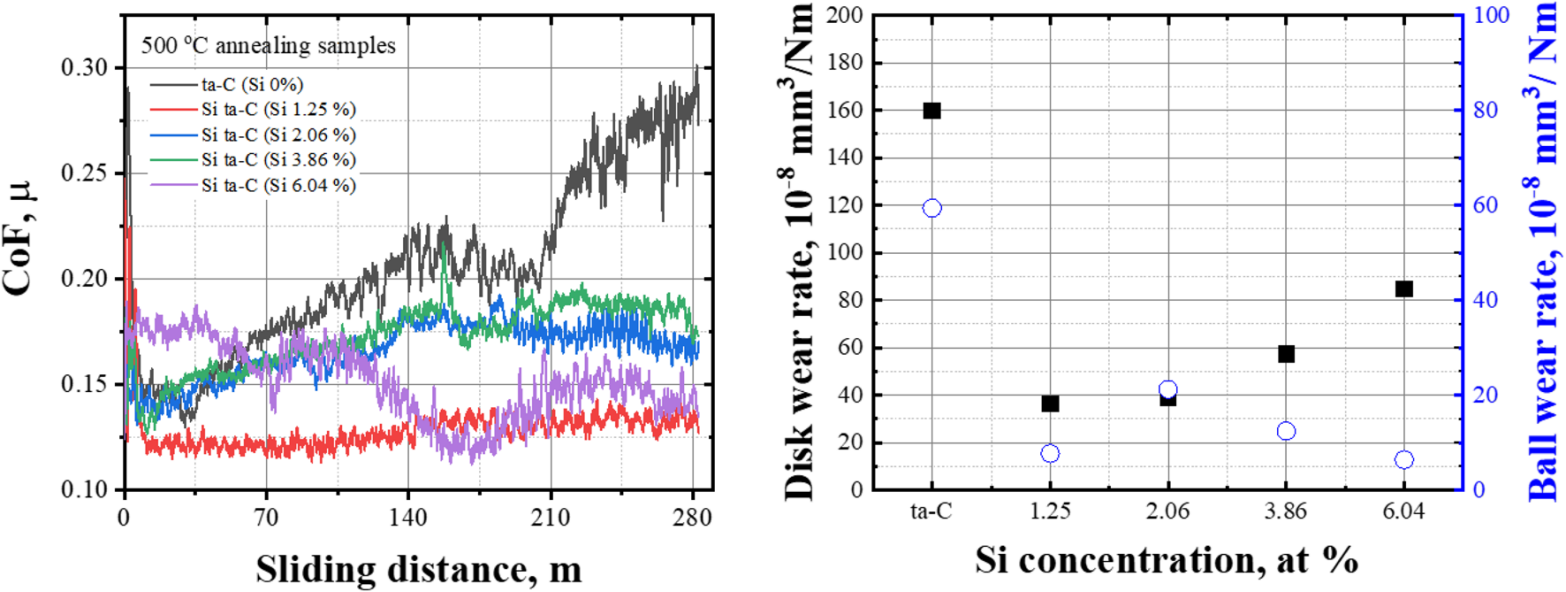
Coefficient of friction (CoF) and disk and Si_3_N_4_ ball wear rate of tetrahedral amorphous carbon (ta-C) and Si/SiC/ta-C composite (Si–ta-C) coatings after annealing at 500 °C.

Figure 13 shows the frictional behavior and wear rate of the disk and ball after annealing at 500 °C. The CoF of the ta-C coating film increased linearly with increasing sliding distance. It was determined that most of the ta-C coating layer and Ti interlayer decreased in weight after annealing owing to the endothermic reaction, exposing the WC–Co substrate. This can be observed in the thermal stability results in Fig. 5. In contrast, the Si–ta-C coating film with a Si concentration ratio of 1.25 at.%—which had the highest hardness (40 GPa) and lowest CoF of the Si–ta-C coatings—exhibited the lowest disk and counterpart wear rates. As the Si concentration increased, an increase in hardness led to unstable friction behavior that caused the wear rate of the disk to increase and the wear rate of the ball to decrease.

In summary, thermal deterioration was confirmed only in ta-C without Si after annealing at 500 °C, and friction-wear properties were enhanced in Si–ta-C, particularly after high-temperature annealing. Thus, the heat-enhanced mechanical properties improved the durability of the coating. This was due to SiC bonding that was achieved by the addition of an appropriate amount of Si. Therefore, the hardness of the ta-C coating film is important to ensure its high-temperature durability. Moreover, the thermal stability and durability characteristics should be matched by co-deposition with a low Si concentration.

## Conclusions

This study established the reliability of DLC coatings upon exposure to high-temperature atmospheric environments after composite with a small amount of Si to improve their thermal degradation and durability while maintaining high mechanical properties. The following results were obtained for the thermal stability and durability tests of the ta-C and Si–ta-C coatings:

1. Si concentration of 1.25–6.04 at.% was achieved by increasing the UBM sputtering power during ta-C coating using FCVA.
2. As the Si concentration increased, the mechanical properties of the Si–ta-C coating were reduced. However, increasing the Si content from 3.85 to 6.04 at.% did not reduce the mechanical properties further (hardness of 24 GPa) owing to an increase in Si–C bonding.
3. Si–ta-C coatings exhibit thermal stability up to 500 and 600 °C, independent of the Si concentration in the examined range. However, at a Si concentration of > 3.86 at.%, the residual Si–ta-C coating after annealing had a thickness of 220 nm, despite a decrease in weight due to high-temperature oxidation.
4. The CoF behavior of the Si–ta-C coating was dominated by the formation of SiO bonds, which exhibited a low energy van der Waals interaction. High-temperature annealing also enabled the activation of the SiO bonds.
5. When the annealing temperature was higher than 300 °C, the wear rate of the disk drastically decreased, and that of its counterpart increased. Thus, the structure of the Si–ta-C coating film was confirmed to change owing to the formation of SiC bonds after annealing at > 300 °C.
6. Thermally induced enhancement of the mechanical and tribological properties was caused by the small addition of Si; Si–ta-C (1.25 at.% Si) exhibited superior friction-wear behavior when annealed at 500 °C.

## Supplementary Information

Supplementary Information.
