# Supplementary material for: Thermal stability of Si/SiC/ta-C composite coatings and improvement of tribological properties through high-temperature annealing
Source: Sci Rep. 2022 Mar 3;12:3536. doi: 10.1038/s41598-022-07514-8 (PMC8894414; doi:10.1038/s41598-022-07514-8)
Supplement: Supplementary file 1 — Supplementary Information. [file 41598_2022_7514_MOESM1_ESM.docx]

**Thermal stability of silicon-doped tetrahedral amorphous diamond-like carbon coatings and improvement of tribological properties through high-temperature annealing**

Young-Jun Jang^1^*, Jae-Il Kim^2^, Won-seok Kim^1^, Do-hyun Kim^1^, Jongkuk Kim^1^

^1^*Department of Extreme Environmental Coatings, Surface Technology Division, Korea Institute of Materials Science, 797, Changwon-daero, Seongsan-gu, Changwon-si, Gyeongsangnam-do 51508, Republic of Korea.*

^2^*Department of Micro-Nano Mechanical Science and Engineering, Graduate School of Engineering, Nagoya University, Furo-cho, Chikusa-ku, Nagoya, Aichi 464-8603, Japan*.

^*^Corresponding Author:

Dr. Young-Jun Jang

Tel: +82-55-294-9501; Fax: +82-55-280-3333

Email: [yjjang@kims.re.kr](mailto:yjjang@kims.re.kr)

**Contents**

**Section S1.** Cross-sectional transmission electron microscopy and energy dispersive X-ray spectroscopy images of the Si-taC coatings produced at a sputtering powers of 25 W and 125 W.

**Section S2.** The fitting of high resolution C1s spectra of the ta-C and Si-taC coatings as a function of sputtering power

**Section S3.** The force-displacement curves for ta-C and Si-taC (1.25 at.%) coatings before and after annealing as a function of temperature

**Section S4.** Wear scar marks for ta-C and Si-taC coating (1.25 at.%) after tribo-test in air

**Section S1.**

**
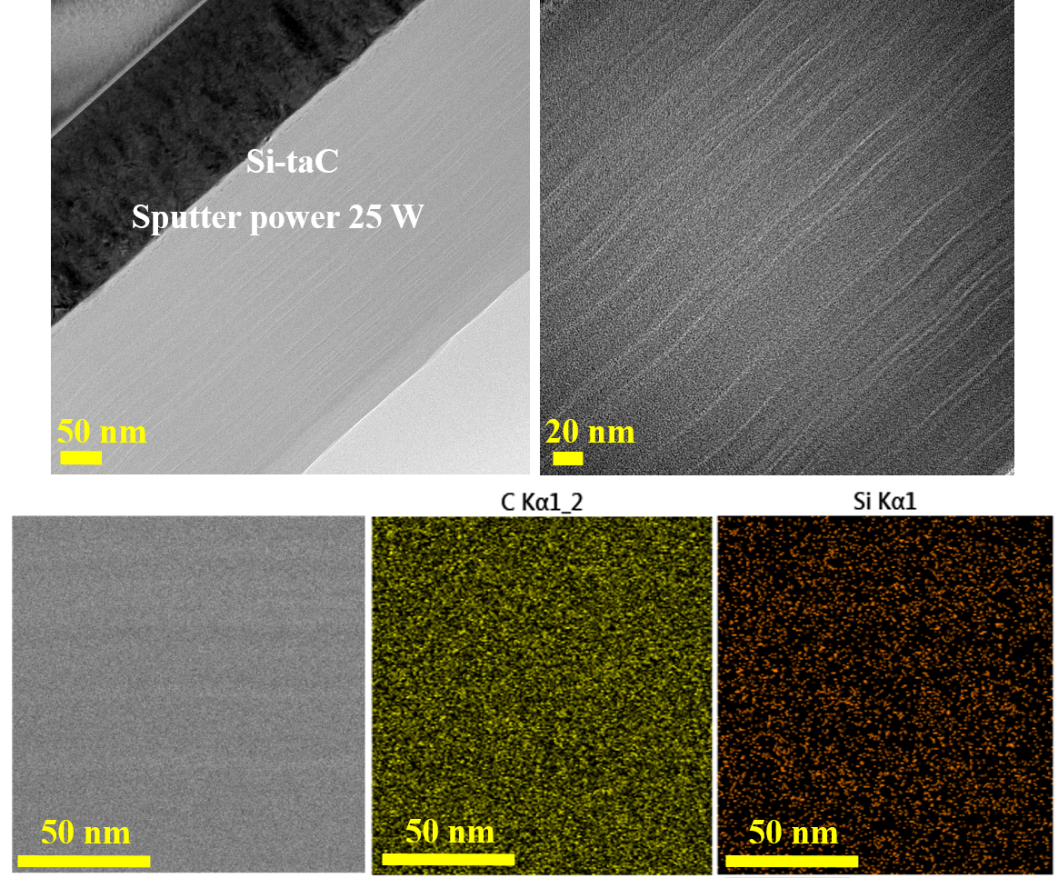
**

**
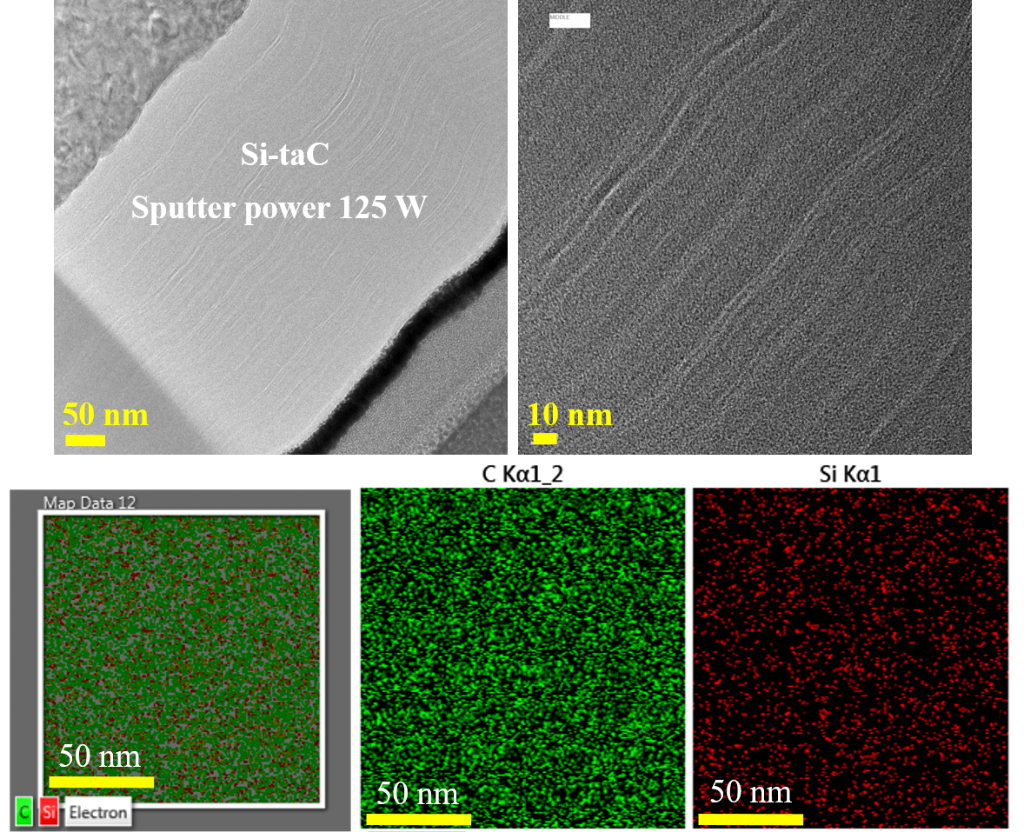
**

**Section S2.**

1. ta-C

1. Si-ta-C (25W)

1. Si-ta-C (75W)

1. Si-ta-C (125W)

1. Si-ta-C (175W)

**Section S3.**


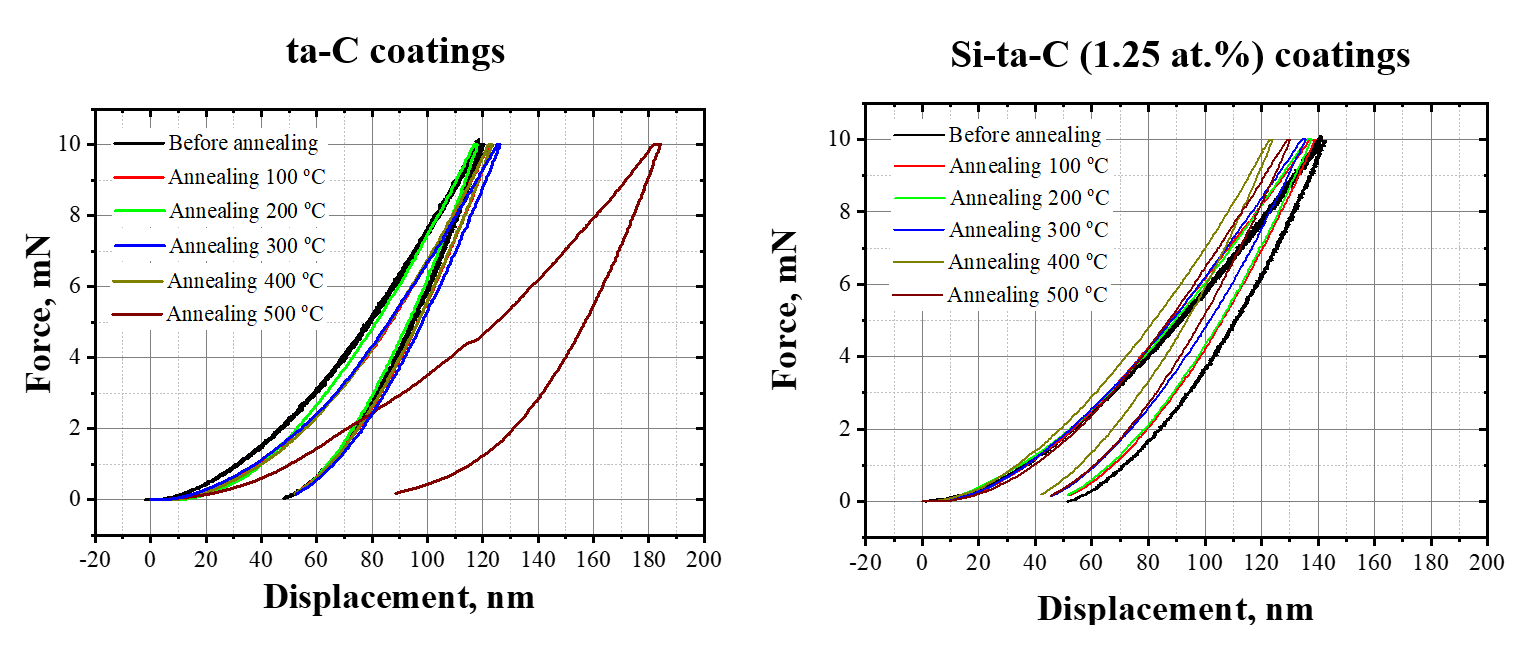


**Section S4.**

**
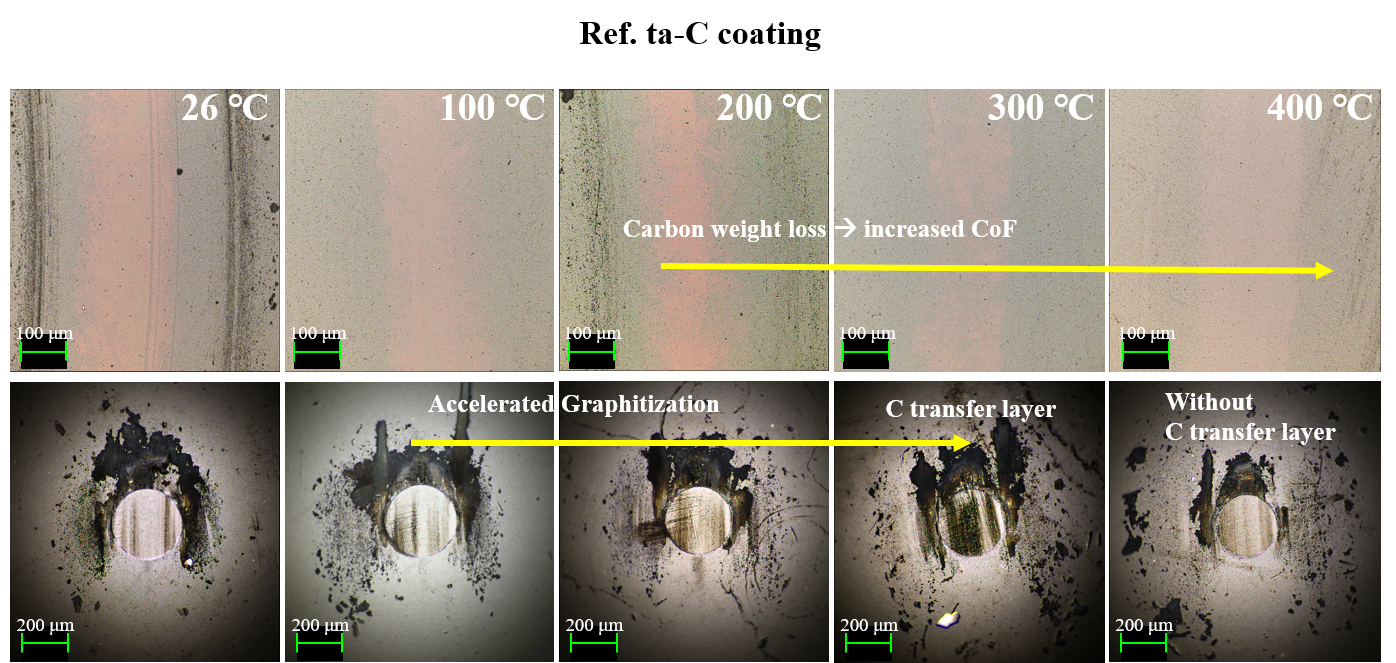
**

**
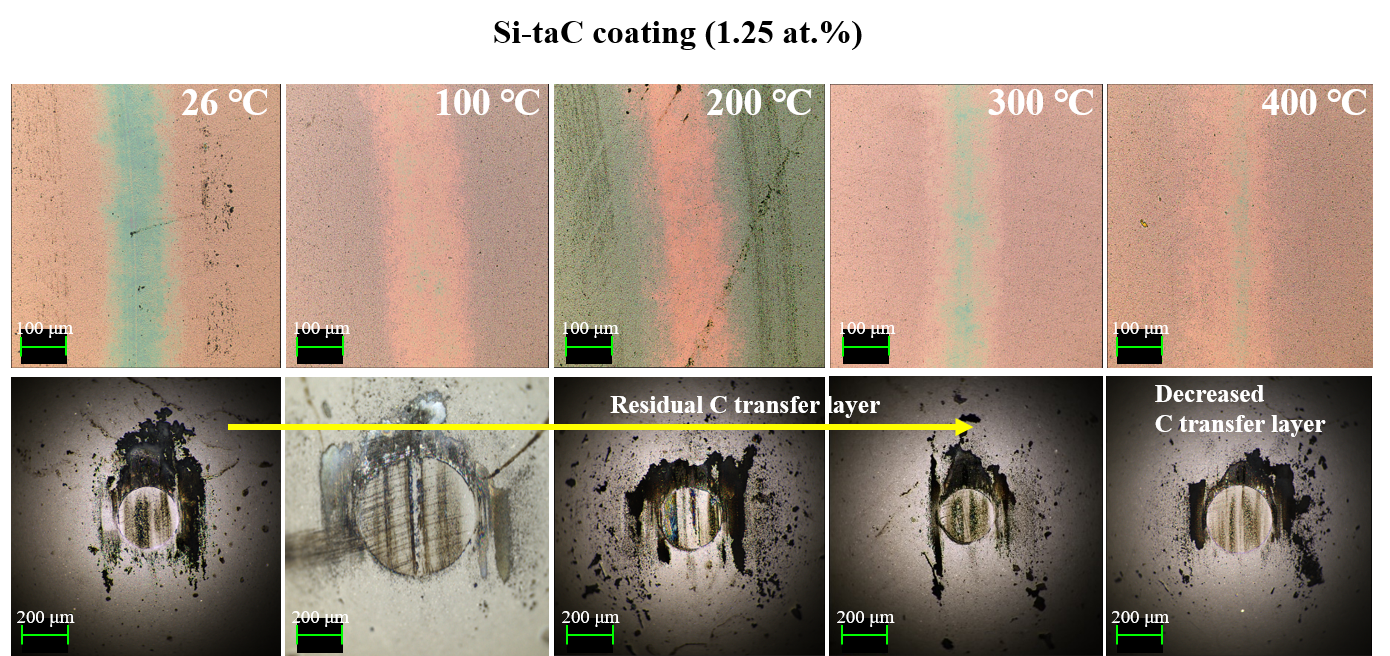
**
